# Preparation of genetically or chemically engineered exosomes and their therapeutic effects in bone regeneration and anti-inflammation

**DOI:** 10.3389/fbioe.2024.1329388

**Published:** 2024-01-19

**Authors:** Xinyue Wang, Weitao Gong, Rongrong Li, Lin Li, Jing Wang

**Affiliations:** ^1^ School of Stomatology, Lanzhou University, Lanzhou, China; ^2^ Clinical Research Center for Oral Diseases, Lanzhou, China

**Keywords:** engineering exosomes, bone regeneration, anti-inflammation, genetically engineered, chemical modification

## Abstract

The treatment of bone or cartilage damage and inflammation-related diseases has been a long-standing research hotspot. Traditional treatments such as surgery and cell therapy have only displayed limited efficacy because they can’t avoid potential deterioration and ensure cell activity. Recently, exosomes have become a favorable tool for various tissue reconstruction due to their abundant content of proteins, lipids, DNA, RNA and other substances, which can promote bone regeneration through osteogenesis, angiogenesis and inflammation modulation. Besides, exosomes are also promising delivery systems because of stability in the bloodstream, immune stealth capacity, intrinsic cell-targeting property and outstanding intracellular communication. Despite having great potential in therapeutic delivery, exosomes still show some limitations in clinical studies, such as inefficient targeting ability, low yield and unsatisfactory therapeutic effects. In order to overcome the shortcomings, increasing studies have prepared genetically or chemically engineered exosomes to improve their properties. This review focuses on different methods of preparing genetically or chemically engineered exosomes and the therapeutic effects of engineering exosomes in bone regeneration and anti-inflammation, thereby providing some references for future applications of engineering exosomes.

## 1 Introduction

**FIGURE 1:**
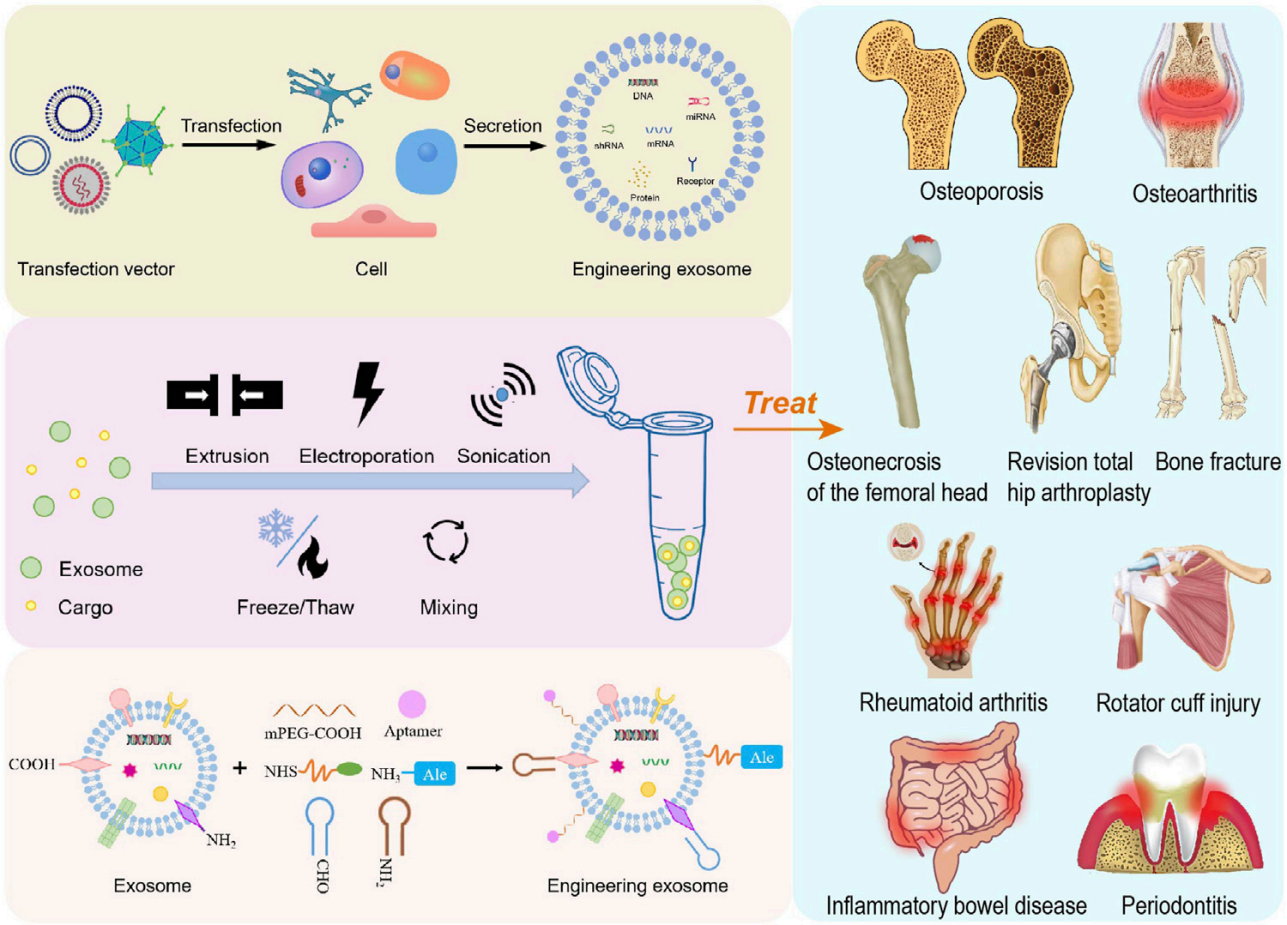
Schematic overview of the preparation of engineering exosomes for the treatment of bone or cartilage damage and inflammation-related diseases.

The treatment of bone or cartilage injury and inflammatory diseases has always been the focus of research, and the present evidence has indicated that systematic bone loss is commonly complicated with chronic inflammation. Osteoporosis (OP) is a chronic and disabling disease which has two characteristics of low bone mass and increased risk of fractures (Liu et al., 2021b). Damage to cartilage causes osteoarthritis (OA), which leads to synovium and meniscus degeneration, subchondral osteosclerosis and aseptic inflammation, resulting in chronic pain and reduced mobility (Felson, 2009; Disease et al., 2017). Osteonecrosis of the femoral head (ONFH) is a refractory and progressive orthopedic disease (Bose and Baruah, 2010). Current treatments for pre-collapsed ONFH are ineffective due to insufficient prevention of compromised subchondral microcirculation, endothelial dysfunction, and inadequate bone repair (Weinstein et al., 2017). As a result, helpful strategies are required to promote osteogenesis and angiogenesis in the early stages of ONFH. Bone fracture healing is a complicated repair procedure, during the early stages of bone fracture healing, inflammatory and immune cells recruited from nearby areas or the circulation congregate at the fracture site (Sabate-Bresco et al., 2021; Shin et al., 2021). A moderate inflammatory response at the early stages of fracture repair is required for proper bone healing, but chronic and overactive inflammation impairs fracture healing. Despite plenty of anti-inflammation drugs develop rapidly over the past few decades, drug overdose and systemic adverse responses continue to be major problems (Wei et al., 2017). Certainly, there are more than a few bone and inflammatory diseases in urgent need of treatment. Currently, conventional treatments for bone or cartilage injury and inflammatory-related diseases typically involve three types: non-pharmacological, pharmacological and surgical therapies. However, there are many issues, including patient compliance, adverse effects of medications and surgical complications during treatment. More importantly, temporary relief of disease-related symptoms rather than bone or cartilage repair and regeneration, occurs in most cases, such that the overall effect of the treatment is unsatisfactory because the progression of diseases is not reversed. In regard to the therapy of these diseases, the common problems need to be solved include bone regeneration and anti-inflammation, so it is imperative to develop safer and more efficient preventive and treatment strategies (Figure 1).

**FIGURE 2:**
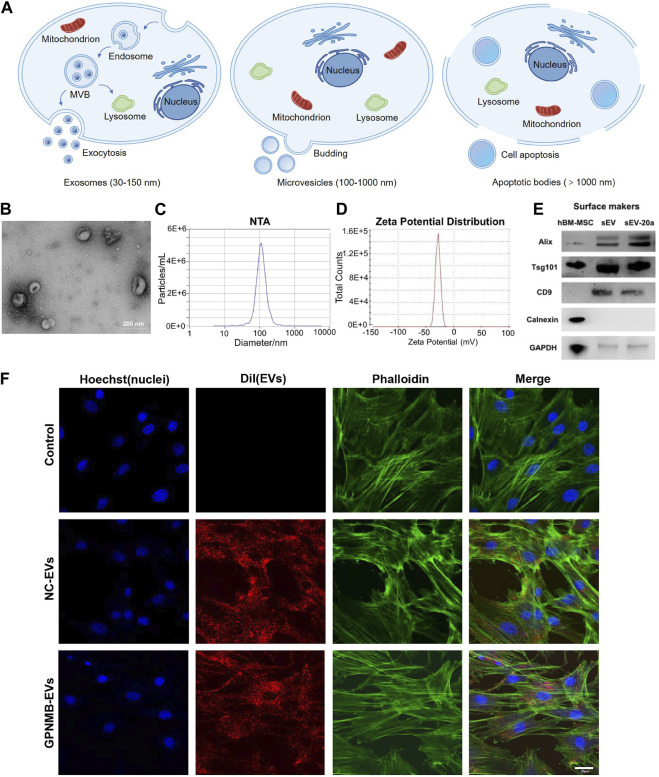
Formation process and characterization of EVs. **(A)** EVs are divided into three types according to their sizes and formation mechanism: exosomes, microvesicles and apoptotic bodies. **(B)** TEM image of EVs derived from HUVEC (Guo B. et al., 2022). **(C)** Nanoparticle tracking analysis (NTA) show that particles size distribution of EVs (Zha et al., 2021). **(D)** Zeta potential distribution of EVs (Zha et al., 2021). **(E)** Western blotting analysis of EVs markers Alix, Tsg101, CD9 and Calnexin (Liu et al., 2021b). **(F)** The uptake of EVs by BMSCs observed by confocal microscopy. Scale bar: 30 µm (Huang et al., 2021).

With the development of regenerative medicine and the safety concerns regarding the toxicity, distribution and potential tumorigenicity of mesenchymal stem cells (MSC), extracellular vesicles (EVs) have been a novel strategy for treating these diseases. EVs are lipid bilayer vesicles secreted by almost all types of cells and reach the whole body through blood circulation (Toh et al., 2017). EVs can be divided into three main types according to their different sizes and formation mechanism: exosomes (exo, 30–150 nm in diameter), microvesicles (MVs, 100–1,000 nm in diameter), and apoptotic bodies (>1,000 nm in diameter) (Figures 2A–D) (Ingato et al., 2016; Elsharkasy et al., 2020). Endocytosis of the cell membrane results in the production of early endosomes, which bud inward to form multivesicular bodies. Then, multivesicular bodies fuse with cell membrane to release exosomes. Microvesicles are obtained by efflux of cell membranes. Apoptotic bodies are formed by cell shrinkage or division as a result of apoptosis. It’s difficult to distinguish exosomes and microvesicles completely because both of them carry a portion of parental cargos and overlap in size. However, apoptotic bodies often have larger particle sizes and contain organelles, which are easier to distinguish from the former two (van Niel et al., 2018; Xu et al., 2019; Mathieu et al., 2019; Kalluri and LeBleu, 2020; Zhao et al., 2021). It's worth noting that EVs in most studies refer to exosomes, which usually appear as round or cup-shaped under the transmission electron microscope (TEM) (Kibria et al., 2016). Exosomes contain a variety of substances including proteins, lipids, DNA, RNA, etc., among which proteins include tetraspanins CD9, CD63, CD81, biogenesis associated protein TSG101, heat shock protein HSP70, metabolic enzyme GAPDH, cell adhesion proteins Integrins and so on (Figure 2E) (Su et al., 2017; Dong et al., 2019; Liang et al., 2019; Chen et al., 2022). Exosomes release contents by fusing with target cell membranes or go into the cytoplasm through endocytosis for release, and they can also activate intracellular signaling cascades by binding to target plasma membrane receptors through surface proteins (Feng et al., 2010; Mulcahy et al., 2014; Yáñez-Mó et al., 2015).

Exosomes from different cells have different functions or characteristics, especially the regeneration capacities of exosomes are susceptible to MSC sources. The positive roles of exosomes secreted by MSC (MSC-exo) have been proven in the repairment and reconstruction of multiple tissues, including skeleton, cartilage and skin (Duan et al., 2020; Hu et al., 2021; Liu et al., 2021). Recent studies have revealed MSC-exo are able to regulate osteogenic differentiation, promote bone regeneration and ameliorate osteopenia *in vivo* (Liu et al., 2015; Qi et al., 2016). Allogeneic MSC-exo-based therapeutics are currently being assessed by early phase clinical trials in regenerative and anti-inflammatory applications (Mendt et al., 2019). A part of exosomes derived from immune cells have also been developed as specific delivery systems due to their inherent targeting ability. Exosomes are characterized by detecting the expression of exosome-specific markers, and common markers of MSC-exo are CD9, CD63, CD81 (Tao et al., 2017d; Chen et al., 2019; Zhang et al., 2020b), TSG101 (Tao et al., 2017b; Luo et al., 2019a), Alix (Tao et al., 2017b; Tao et al., 2017d), Calnexin (Nan et al., 2021b) and Flotillin 1 (Zarubova et al., 2022). It is worth noting that there are some special examples. Exosomes from osteoclasts and their precursors contain the exosomal markers EpCAM and CD63 and lack gp96 and Calnexin, endoplasmic reticulum proteins that are often found in contaminated exosome preparations (Huynh et al., 2016). One study has shown that both BMSC-exo and GMSC-exo express exosome-specific markers, but the expression levels of CD9 and CD81 are different, with BMSC-exo containing lower levels of both markers (Zarubova et al., 2022).

Exosomes have many advantages, such as low immunogenicity, good biocompatibility, low side effects, high stability in the circulation and efficient intercellular communication when compared with cell or nanoparticle therapy (Johnsen et al., 2018). Although exosomes have their own advantages in tissue regeneration, they still have disadvantages such as low yield, low targeting and weak function, which can’t meet the requirements of quantity and quality of disease treatment. Therefore, a series of methods need to be adopted to develop engineering exosomes to overcome the limitations of natural exosomes (Cheng et al., 2022). This review summarizes the research progress of genetically or chemically engineered exosomes in bone regeneration and anti-inflammation in recent years, and provides certain references for the development of engineering exosomes with better therapeutic effects in the future.

## 2 Different methods for preparation of engineering exosomes

**FIGURE 3:**
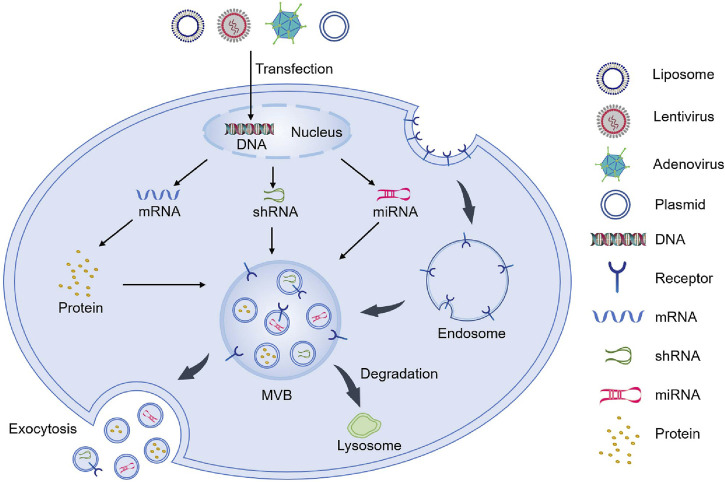
Endogenous loading methods to prepare genetically engineered exosomes.

**FIGURE 4:**
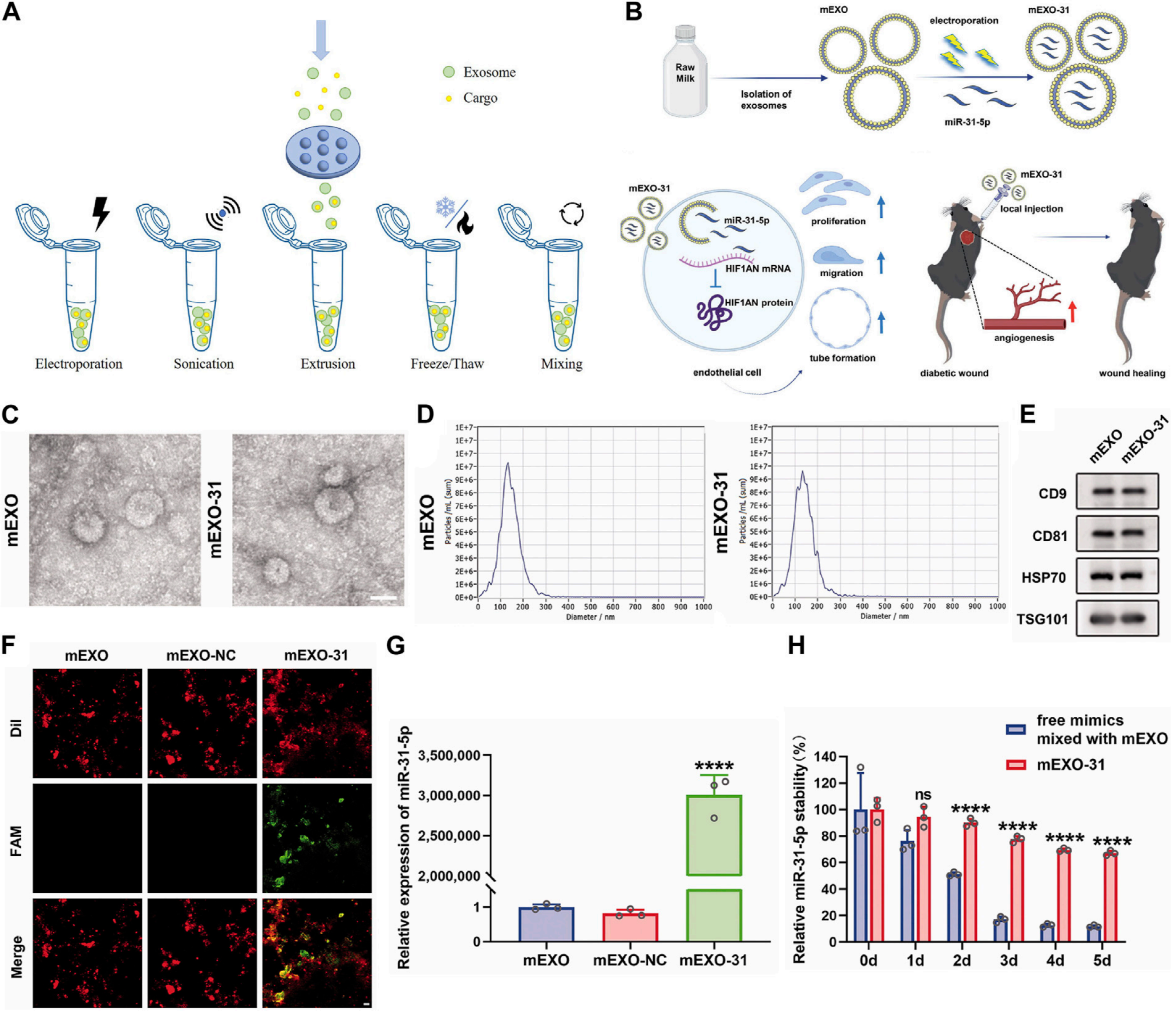
Exogenous loading methods to prepare engineering exosomes. **(A)** Exogenous loading methods include electroporation, sonication, extrusion, freeze/thaw and mixing. **(B)** Isolation of milk-derived exosomes (mEXO) and preparation of miR-31-5p-loaded exosomes (mEXO-31) by electroporation, action mechanism of mEXO-31 *in vitro* and mEXO-31 treatment on mice model (Yan et al., 2022). **(C)** TEM identified the morphology of mEXO and mEXO-31. Scale bar: 50 µm (Yan et al., 2022). **(D)** NTA identified the size distribution of mEXO and mEXO-31 (Yan et al., 2022). **(E)** Western blotting of markers CD9, CD81, HSP70, and TSG101 of mEXO and mEXO-31 (Yan et al., 2022). **(F)** Confocal images showed successful loading of miR-31-5p into mEXO. Red and green fluorescence represented mEXO and miR-31-5p mimics, respectively. Scale bar: 10 µm (Yan et al., 2022). **(G)** RT-PCR analysis of relative miR-31-5p levels in mEXO, milk-derived exosomes loaded with mimic NC (mEXO-NC), and mEXO-31 (Yan et al., 2022). **(H)** RT- PCR analysis of remaining miR-31-5p in each group (Yan et al., 2022).

At present, lots of researchers load cargos into exosomes to prepare genetically engineered exosomes to improve their properties and enhance the therapeutic effects of diseases. Loading methods are mainly divided into two categories: endogenous and exogenous loading methods (Elsharkasy et al., 2020; Soekmadji et al., 2020). The endogenous loading method refers to the genetically engineered cells that are modified by transgenic technology to secrete genetically engineered exosomes with the similar characterization. There are some familiar transfection vectors: lentivirus, plasmid, liposome and so on (Wang et al., 2018b; Hong et al., 2018; Zhang et al., 2018; Jin et al., 2020; Pan et al., 2020; Zhong et al., 2021; Liu et al., 2022). The target genes can be encapsulated into exosomes through intracellular cargo sorting mechanism (Figure 3; Table 1). The exogenous loading method refers to direct loading target genes or other cargos into exosomes through physical methods to prepare genetically engineered exosomes. The frequent physical methods include: co-incubation, electroporation, sonication, mechanical extrusion, etc (Figure 4A) (Haney et al., 2015). All of these methods can deliver cargos to exosomes such as miRNA, siRNA, proteins, lipophilic drugs like curcumin (Cur) (Lin et al., 2018; Wang et al., 2019; Guo et al., 2019; Pomatto et al., 2019; He et al., 2020; Wang et al., 2021), and water-soluble drugs like berberine (Table 2) (Dong et al., 2022). Therapeutic miRNA and proteins are usually loaded into exosomes using endogenous loading methods, while small molecule cargos are mainly loaded using exogenous loading methods, sometimes endogenous and exogenous methods can be combined to produce engineering exosomes. In addition, chemical modification to prepare engineering exosomes can achieve targeting or better therapeutic effects, is also widely developed and adopted (Table 3).

**TABLE 1:** Genetically engineered exosomes prepared by endogenous methods and origin of exosomes.

Origin of exosomes	Modification	Transfection vectors	Applications	References
BMSC	miR-29a	Liposome	Promote osteogenesis and angiogenesis and treat OP	Lu et al. (2020)
BMSC	miR-20a	Liposome	Enhance osteogenesis and treat osteoporotic bone defects	Liu et al. (2021b)
BMSC	miR-150-5p	Lentivirus	Reduce bone resorption, increase bone mass and treat diabetic osteoporosis	Xu et al. (2022a)
BMSC	miR-140-3p	Lentivirus	Alleviate bone degradation, promote bone restoration and treat diabetic-associated impaired bone healing	Wang et al. (2022)
BMSC	miR-181b	Liposome	Inhibit inflammation and promote osteogenesis and osteointegration	Liu et al. (2021c)
SMSC	miR-140-5p	Lentivirus	Enhance cartilage tissue regeneration and prevent osteoarthritis	Tao et al. (2017c)
SMSC	miR-126-3p	Lentivirus	Accelerate epithelial cell re-epithelialization, promote angiogenesis and collagen maturation and heal full-thickness skin defect	Tao et al. (2017a)
ADSC	miR-375	Lentivirus	Promote bone regeneration	Chen et al. (2019a)
ADSC	miR-378	Liposome	Promote angiogenesis and osteogenesis and treat ONFH	Nan et al. (2021a)
BMSC	BMP-2	Liposome	Promote bone regeneration	Huang et al. (2020), Li et al. (2022a)
BMSC	NELL1	Lentivirus	Promote BMSC osteogenesis and bone regeneration	Lan et al. (2022)
HUVEC/BMSC	PD-L1	Liposome/Lentivirus	Promote osteogenic differentiation and fracture healing/Reconfigure local immune microenvironment	Xu et al. (2022b), Lin et al. (2022)
NIH-3T3/MC3T3	CXCR4	Lentivirus/Lentivirus	Promote osteogenesis, reverse age-related trabecular bone loss and decrease cortical bone porosity/Enhance M2 macrophage polarization, combat inflammation and improve targeting ability	Hu et al. (2021b), Wang et al. (2021)
NIH-3T3	GLG1	Lentivirus	Alleviate bone loss, promote bone formation and accelerate fracture healing	Guo et al. (2022b)
HEK293T	TNFR1	Lentivirus	Ameliorate inflammatory disease phenotypes	Gupta et al. (2021)
HEK293T	IL-6ST	Lentivirus	Ameliorate inflammatory disease phenotypes	Gupta et al. (2021)
DC	E7	Plasmid	Target tumor cells, myocardial cells and SMSC	Shao et al. (2012), Meng et al. (2015), Meng et al. (2017), Xu et al. (2021)
HEK293FT	NFIC	Lentivirus	Promote proliferation, migration and odontogenic differentiation of apical papilla and dentin formation	Yang et al. (2022)
BMSC	Scx	Lentivirus	Inhibit osteolysis, reduce osteoclast and enhance tendon-bone healing strength	Feng et al. (2021)
BMSC	GPNMB	Lentivirus	Stimulate BMSC proliferation and osteogenic differentiation and attenuate bone loss in OP	Huang et al. (2021)
PDLSC	P2X7R	Adenovirus	Reverse inflammation-mediated impairment of PDLSC and promote tissue regeneration	Xu et al. (2020a)
BMSC	Noggin-shRNA	Lentivirus	Promote bone regeneration	Fan et al. (2020)
BMSC	SMURF1-shRNA	Lentivirus	Enhance osseointegration	Xu et al. (2022c)

**TABLE 2:** Engineering exosomes prepared by exogenous methods and origin of exosomes.

Origin of exosomes	Modification	Methods	Applications	References
MC3T3-E1	Let-7	Electroporation	Regulate osteogenic differentiation	Choi et al. (2019)
Milk	miR-31-5p	Electroporation	Improve endothelial cell function and enhance the healing process of diabetic wound	Yan et al. (2022)
J774A.1	BMP-2	Electroporation or sonication	Induce osteogenesis	Yerneni et al. (2021)
ATDC5	VEGF	Electroporation	Enhance osteogenic induction and vascular remodeling in large segmental bone defects	Zha et al. (2020), Zha et al. (2021)
iPS-derived MSC	Shn3-siRNA	Electroporation	Enhance osteogenic differentiation and promote the formation of blood vessels	Cui et al. (2022)
BMSC	FGF2-siRNA	Electroporation	Improve proliferative ability of vascular endothelial cells and promote ONFH repair	Zhang et al. (2020a)
BMSC	Wnt-11-siRNA	Electroporation	Improve proliferative ability of vascular endothelial cells and promote ONFH repair	Zhang et al. (2020a)
BMSC	S100A9-siRNA	Electroporation	Improve proliferative ability of vascular endothelial cells and promote ONFH repair	Zhang et al. (2020a)
BMSC	FSTL1-siRNA	Electroporation	Improve proliferative ability of vascular endothelial cells and promote ONFH repair	Zhang et al. (2020a)
BMSC	TNF-α-siRNA	Electroporation	Improve proliferative ability of vascular endothelial cells and promote ONFH repair	Zhang et al. (2020a)
BMSC	Caspase3-siRNA	Electroporation	Improve proliferative ability of vascular endothelial cells and promote ONFH repair	Zhang et al. (2020a)
Macrophage	MVs-coated PLGA	Sonication	Target RA and suppress RA progression	Li et al. (2019b)
BMSC	PASP-PLGA	Co-incubation	Target BMSC and promote tendon-bone healing	Han et al. (2022)

**TABLE 3:** Engineering exosomes prepared by chemical methods and origin of exosomes.

Origin of exosomes	Modification	Methods	Applications	References
ADSC	CREKA	Hydrophobic insertion method	Target fibrin–fibronectin complexes, promote osteogenic differentiation and angiogenesis and enhance bone repair	Wu et al. (2023)
Schwann cell	Phosphatidylserine-targeting aptamer	Condensation reaction	Induce nerve, blood vessel and bone regeneration	Ashrafuzzaman et al. (2013), Su et al. (2022)
Bioinspired EVs	Osteoblast-targeting aptamer	Condensation reaction	Target osteoblast and promote bone regeneration	Shao et al. (2016), Wang et al. (2019b)
BMSC	Bone marrow-targeting aptamer	Schiff base reaction	Promote bone regeneration, accelerate bone healing and treat OP and fracture	Luo et al. (2019b)
GMSC	PLGA	EVs are immobilized on the surface of the PLGA via a matrix metalloproteinases (MMPs)-sensitive linker	Decrease the secretion of pro-inflammatory cytokines, suppress T cells activation and improve regeneration of damaged periodontal tissue	Zarubova et al. (2022a)
ADSC	DS	Click chemistry	Induce anti-inflammatory responses	You et al. (2021)
Mouse MSC	Ale	Click chemistry	Target bone and treat OP	Wang et al. (2020)

## 3 Genetically engineered exosomes prepared by endogenous methods

### 3.1 Genetically engineered exosomes modified with miRNA

MicroRNA (miRNA) are small non-coding RNA and involve in almost all cellular processes as mediators of mRNA translational efficiency (Li et al., 2015). Recently, part of miRNA have been demonstrated to be involved in bone metabolism and osteogenic regulation and are important for bone remodeling such as angiogenesis and osteogenesis (Lian et al., 2012; Feng et al., 2018; Zhao et al., 2018; Nan et al., 2021a). Thus, miRNA are applied to improve local bone quality and can also be incorporated into biocompatible scaffolds for tissue regeneration (Haussecker, 2014; Raftery et al., 2016). Despite the high potential of miRNA-based gene therapy, it has many obstacles that need to be overcome. Firstly, due to the negative charge of miRNA, they are difficult to pass through cell membranes (Boca et al., 2020). In addition, miRNA are unstable and tend to be easily degraded by RNase before entering target cells and have a short half-life *in vivo*, which limits their application in bone tissue engineering (O'Brien et al., 2018). Therefore, the development of safe and efficient miRNA delivery systems is of great significance to optimize miRNA-based gene therapy. With the development of cell-free transplantation strategy, exosomes can be used as a carrier of osteogenic miRNA to achieve a combination of their functions and effects.

#### 3.1.1 Bone marrow mesenchymal stem cells-derived exosomes

Previous research suggested that miR-29a level was high in the exosomes from bone marrow mesenchymal stem cells (BMSC), which can be transported into human umbilical vein endothelial cells (HUVEC) to regulate angiogenesis, and VASH1 was identified as a direct target of miR-29a. However, the miR-29a level was dramatically decreased in aged BMSC-exo compared to young BMSC-exo, thus miR-29a mimics were transferred into aged BMSC to collect the miR-29a-loaded BMSC-exo (BMSC-miR-29a-exo). BMSC-miR-29a-exo showed robust ability of promoting osteogenesis and angiogenesis *in vivo* and may serve as a potential therapeutic target for OP (Lu et al., 2020). The miR-20a could enhance osteogenesis and EVs derived from BMSC could be utilized as nanoscale carriers for the protection and transportation of miR-20a. EVs overexpressing miR-20a (BMSC-miR-20a-EVs) exerted a superior effect on osteoporotic bone defects and effectively promoted osteoporotic porous titanium alloy osteointegration via pro-osteogenic effects by targeting BAMBI (Liu et al., 2021b). miR-150 has the ability to induce the osteoblastic phenotype, which is associated with osteoblast function and bone mineralization (Dong et al., 2015). In particular, miR-150-5p has been identified to target matrix metalloproteinase 14 (MMP14) and inhibition of MMP14 can reduce bone resorption and increase bone mass (Delgado-Calle et al., 2018). Researchers constructed BMSC-miR-150-5p-EVs to deliver miR-150-5p to osteoblasts where miR-150-5p targeted MMP14 and consequently activated Wnt/β-catenin pathway. Then, BMSC-miR-150-5p-EVs were loaded on gold-coated magnetic nanoparticles (GMNP) and N52 neodymium magnet was used to provide external magnetic field on rat femur, with the flowing GMNP-EVs gathered to the femur. In short, this work suggested the potential of engineering exosomes to strengthen osteoblast proliferation and maturation in diabetic osteoporosis, offering a desirable drug delivery strategy to combat diabetic osteoporosis (Xu et al., 2022). Moreover, BMSC were transfected with lentivirus that overexpressed miR-140-3p to obtain exosomes overexpressing miR-140-3p (BMSC-miR-140-3p-exo). BMSC-miR-140-3p-exo transplanted into the bone defects alleviated bone degradation and promoted bone regeneration by targeting the plexin B1/RohA/ROCK signaling pathway in diabetic rats, which offered a new insight for treating diabetic-associated impaired bone healing (Wang et al., 2022). Exosomes from BMSC overexpressing miR-181b (BMSC-miR-181b-exo) displayed beneficial effects on promoting M2 macrophage polarization, inhibiting inflammation as well as promoting osteogenesis. To be specific, BMSC-miR-181b-exo suppressed inflammatory response by promoting M2 polarization via activating PRKCD/AKT signaling pathway, which further promoting osteogenesis *in vitro* and osseointegration *in vivo* (Liu et al., 2021c).

#### 3.1.2 Synovial mesenchymal stem cells-derived exosomes

Engineering exosomes (SMSC-miR-140-5p-exo) derived from miR-140-5p-overexpressing synovial mesenchymal stem cells (SMSC) enhanced the proliferation and migration abilities of articular chondrocytes without harming extracellular matrix secretion of articular chondrocytes. It has been proved that Wnt5a and Wnt5b carried by exosomes activated Yes-associated protein via the alternative Wnt signaling pathway and enhanced proliferation and migration of chondrocytes with the side-effect of significantly decreasing extracellular matrix secretion, however, SMSC-miR-140-5p-exo could block this side-effect via RalA and prevent OA in rat models (Tao et al., 2017c). In addition, SMSC can stimulate fibroblast proliferation but not angiogenesis. Transgenic technology was used to overexpress miR-126-3p in SMSC, which transferred the angiogenic ability of endothelial progenitor cells into SMSC to promote angiogenesis. In a dose-dependent manner, transgenic SMSC secreted miR-126-3p exosomes (SMSC-miR-126-3p-exo) to stimulate the proliferation of human dermal fibroblasts and the proliferation, migration, and luminal formation of human dermal microvascular endothelial cells. It was known that chitosan hydrogel was a hemostatic, antibacterial, biodegradable, and biocompatible transporter for the sustained release of nanoparticles (El-Newehy et al., 2015; Shi et al., 2015), SMSC-miR-126-3p-exo wrapped in chitosan hydrogel were used in full-thickness skin defect on the back of diabetic rats, demonstrating the effect of accelerating epithelial cell re-epithelialization and promoting angiogenesis and collagen maturation, which was expected to be a potential strategy for the treatment of diabetic wounds (Tao et al., 2017a).

#### 3.1.3 Adipose-derived stem cells-derived exosomes

Engineering exosomes enriched with miR-375 (ADSC-miR-375-exo) were generated from adipose-derived stem cells (ADSC) overexpressing miR-375 after lentiviral transfection, which could promote bone regeneration. After incorporated with hydrogel, ADSC-miR-375-exo displayed a slow and controlled release and further enhanced the bone regenerative capacity in calvarial defect rat models (Chen et al., 2019). Besides, exosomes derived from miR-378-overexpressing ADSC (ADSC-miR-378-exo) promoted angiogenesis and osteogenesis in GC-induced ONFH. Administration of ADSC-miR-378-Exo enhanced the osteogenic and angiogenic potentials of BMSC and HUVEC by targeting Sufu to upregulate the Shh signaling pathway (Nan et al., 2021a).

### 3.2 Genetically engineered exosomes modified with proteins

#### 3.2.1 Growth factors

Transforming growth factor is a kind of endogenous biological polypeptide, which can stimulate cell growth and differentiation, cartilage synthesis and regeneration of damaged cartilage tissue (Krstic et al., 2018; Shao et al., 2020). Bone morphogenetic protein (BMP), which belongs to the family of transforming growth factors, is the only growth factor that can induce bone tissue formation alone. Among them, BMP-2 is an effective inducing factor for the growth and differentiation of osteogenic and chondrogenic cells and is used in many bone regenerative applications. However, the clinical application of BMP-2 remains challenging, mainly because large pharmacological doses of BMP-2 are required due to the poor binding of BMP-2 to the collagen type I sponge, which results in burst release and short residence time *in vivo*. Therefore, it is necessary to solve the problems of carrier, optimal dose, and effective time of growth factor release in the human body. EVs as delivery vehicles for BMP-2 have several potential advantages: the high binding capacity with extracellular matrix constituents and the ability to protect intraluminal cargos from antagonists, inhibitors or enzyme degradation (Lykissas and Gkiatas, 2017). EVs with enhanced osteoinduction ability (BMSC-BMP2-EVs) were collected from genetically modified BMSC overexpressing BMP-2, and the BMSC-BMP2-EVs showed increased bone regenerative potential compared to BMSC-EVs in rat calvarial defect models. The data indicated that the enhanced regenerative potential of BMSC-BMP2-EVs was due to altered miRNA that amplified the BMP2 signaling cascade (Huang et al., 2020). BMP-2 overexpressing BMSC secreted BMP2-rich exosomes (BMSC-BMP2-exo), which did not affect the morphology, size, markers, and endocytic properties of exosomes, and they could promote osteogenic differentiation safely and effectively and expedite regeneration in both trabecular and cortical bones through BMP2/Smad pathway (Li et al., 2022).

Neural epidermal growth factor-like 1 (NELL1) is a protein that induces bone and cartilage regeneration and outperforms BMP-2 in terms of osteogenic, angiogenic, and anti-inflammatory properties. In various preclinical animal models, NELL1 has shown to be effective in bone regeneration for systemic and local osteogenic treatments (Zhang et al., 2010; Zhang et al., 2014; James et al., 2015). Furthermore, NELL1 significantly activates several pathways, including mitogen activated protein kinase pathway, Wnt/β-catenin pathway and Indian hedgehog pathway, all of which are involved in osteogenesis regulation (Li et al., 2019). EVs derived from NELL1-modified BMSC (BMSC-NELL1-EVs) had a greater ability to promote BMSC osteogenesis due to miR-25-5p downregulation. miR-25-5p inhibited osteogenesis by targeting Smad2 and suppressing the SMAD and extracellular signal-related kinase 1 and 2 pathway activation. In addition, the three-dimensional (3D) BMSC-NELL1-EVs-hydrogel system promoted bone regeneration *in vivo*, which was probably due to a slow, continuous release and high concentration of EVs in the bone defect area. Thus, BMSC-NELL1-EVs might be a novel acellular bone regeneration strategy (Lan et al., 2022).

#### 3.2.2 Receptor and ligand proteins

PD-1 is an important inhibitory receptor, which is primarily expressed on various activated immune cells (including T cells, B cells, macrophages, and dendritic cells etc.,) (Sun et al., 2018). PD-1 interacts with its ligand PD-L1 or PD-L2 to activate immune cells resulting in the state of exhaustion, dysfunction and increased apoptosis, thereby limiting the function of harmful hyperinflammatory responses during chronic infection and autoimmunity (Andrews et al., 2019). PD-L1 specifically binds to PD-1 and transmits inhibitory signal transduction to suppress the T cells proliferation (Xu et al., 2020; Ioannou et al., 2021), which may be a promising target in conditions related to an overactive immune response. Recent research has revealed that HUVEC could inhibit the regulatory cells activation via modulation of PD-L1 expression. Engineering exosomes overexpressing PD-L1 from genetically engineered HUVEC (HUVEC-PD-L1-exo) specifically bound to PD-1 on the T cell surface suppressing the activation of T cells, and also induced MSC toward osteogenic differentiation. HUVEC-PD-L1-exo were embedded in a hydrogel, which allowed exosomes delivery to the surrounding microenvironment in a time-released manner, markedly promoted callus formation and fracture healing in murine models at the early overactive inflammation phase (Lin et al., 2022). Additionally, researchers used lentivirus-mediated gene transfection technology to develop exosomes overexpressing PD-L1 (BMSC-PD-L1-EVs) for reconfiguration of the local immune microenvironment. BMSC-PD-L1-EVs exhibited an impressive ability to regulate various activated immune cells to an immunosuppressed state *in vitro*. More importantly, BMSC-PD-L1-EVs significantly accumulated in the inflamed tissues in dextran sulfate sodium-induced ulcerative colitis and imiquimod-induced psoriasis mouse models, which reshaped the inflammatory ecosystem in the local immune context (Xu et al., 2022).

**FIGURE 5:**
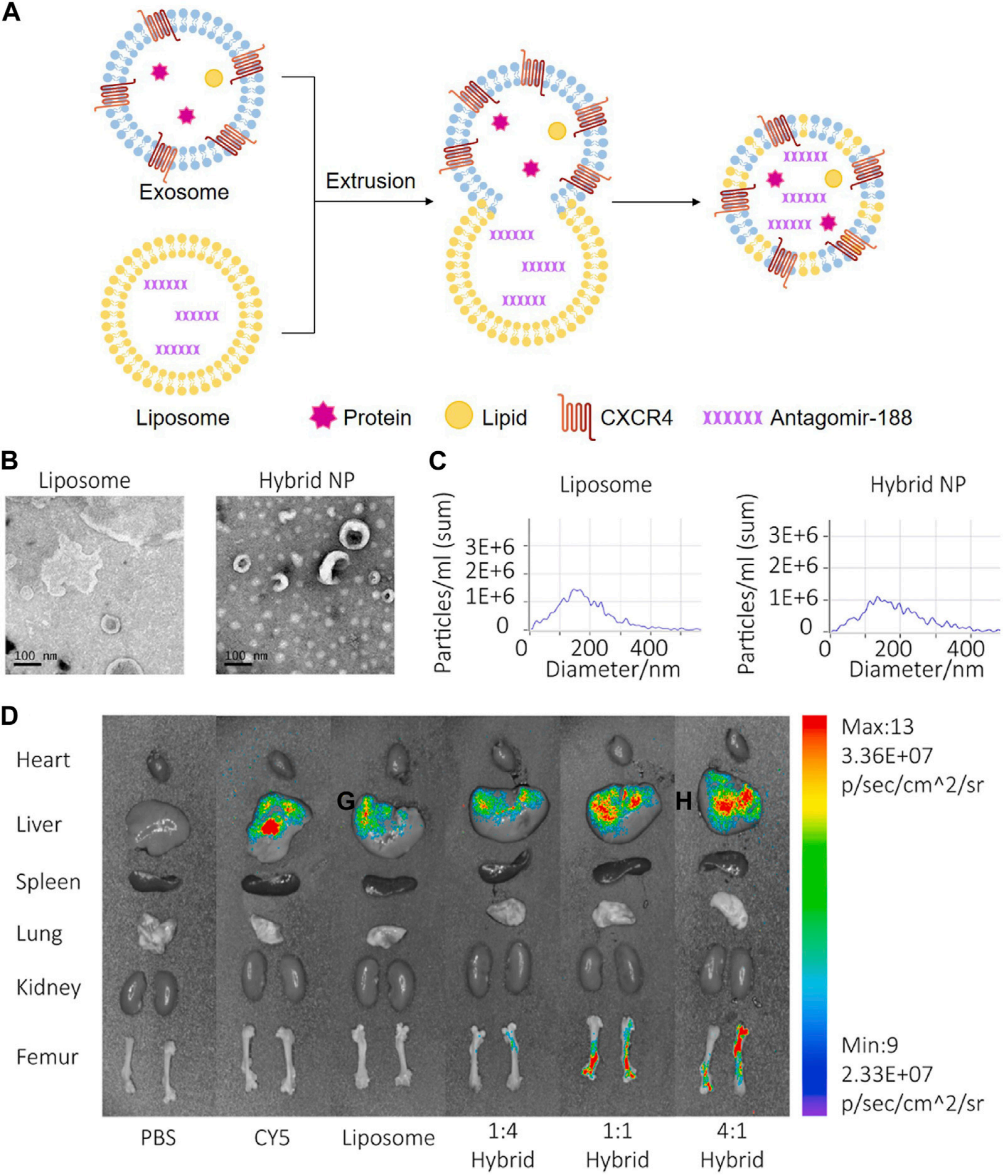
Endogenous and exogenous loading methods are combined to prepare genetically engineered exosomes. **(A)** Display CXCR4 on the surface of exosomes derived from NIH3T3 cells, and then fuse with liposomes carrying antagomir-188 to produce hybrid nanoparticles by extrusion technique. **(B)** TEM of liposome and hybrid nanoparticles (Hu Y. et al., 2021). **(C)** NTA of liposome and hybrid nanoparticles (Hu Y. et al., 2021). **(D)** *In vivo* distribution of CXCR4+ hybrid NPs. Biophotonic images of the organ distribution 4h after intravenous injection of PBS, Cy5, Cy5 labeled liposomes and Cy5-labeled hybrid NPs, with various exosome-liposome ratios (Hu Y. et al., 2021).

Chemokine C-X-C motif ligand 12 (CXCL12), also known as stromal cell-derived factor 1, is primarily expressed by BMSC and functions as a ligand for the chemokine C-X-C motif receptor 4 (CXCR4) (Geminder et al., 2001). CXCL12 constitutes niches of hematopoietic stem cells and regulates hematopoiesis (Zehentmeier and Pereira, 2019). CXCL12 is highly enriched in the bone marrow and specifically bind with CXCR4 for CXCR4-positive hematopoietic stem cells homing. Considering the critical role of the CXCL12/CXCR4 axis in chemotaxis behavior, researchers displayed CXCR4 on the surface of exosomes (NIH3T3-CXCR4-exo) derived from genetically engineered NIH3T3 cells, then fused NIH3T3-CXCR4-exo with liposomes carrying antagomir-188 to produce hybrid nanoparticles by extrusion technique (Figure 5A). The hybrid nanoparticles specifically gathered in the bone marrow and released antagomir-188, which promoted osteogenesis and inhibited adipogenesis of BMSC and thereby reversed age-related trabecular bone loss and decreased cortical bone porosity in mice. It was a novel way to obtain bone-targeted exosomes and a promising anabolic therapeutic approach for age-related bone loss (Figures 5B–D) (Hu et al., 2021). Previous studies have also shown that CXCL12 levels increase markedly in inflammatory tissues and the CXCL12/CXCR4 binding plays an important role in chemotactic processes, such as leukocyte recruitment and migration (Dotan et al., 2010; De Filippo and Rankin, 2018; Janssens et al., 2018). Therefore, EVs enriched CXCR4 (CXCR4-EVs) were obtained from genetically engineered MC3T3 cells, and then Cur, an effective natural anti-inflammatory compound, was encapsulated into CXCR4-EVs through physical entrapment to prepare MC3T3-CXCR4/Cur-EVs. MC3T3-CXCR4/Cur-EVs enhanced M2 macrophage polarization, exhibited anti-inflammatory effects, and significantly improved homing ability via the CXCR4/CXCL12 axis, which could be exploited as a targeting delivery strategy for treatment of inflammatory diseases (Wang et al., 2021).

It’s reported that bone metastasis of cancer cells is mediated by the binding of the ligand Golgi glycoprotein 1 (GLG1) to the receptor E-selectin because E-selectin is usually expressed in the bone vascular niche (Esposito et al., 2019). Thus, researchers constructed GLG1-overexpressed NIH-3T3 cell line and collected GLG1-modified exosomes (NIH-3T3-GLG1-exo) to target the BMSC niche, delivering Wnt agonist 1 to rescue BMSC commitment and promote formation of osteoblastic in inflammatory bowel disease (Guo et al., 2022).

Tumor necrosis factor receptor 1 (TNFR1) and interleukin-6 signal transducer (IL-6ST) could act as decoy receptors for the pro-inflammatory cytokines tumor necrosis factor alpha (TNF-α) and IL-6, respectively. Thus, researchers used cytokine binding domains derived from TNFR1 and IL-6ST to display these two different protein receptors on EVs derived from HEK293T (HEK293T-TNFR1-IL-6ST-EVs). In mouse models of systemic inflammation, neuroinflammation and intestinal inflammation, EVs displaying the cytokine decoys ameliorated the disease phenotypes with higher efficacy as compared with clinically approved biopharmaceutical agents targeting TNF-α and IL-6 pathways (Gupta et al., 2021).

The lysosome-associated membrane glycoprotein 2b (Lamp2b) was a protein specifically expressed on exosome membrane, which can be genetically modified to induce engineering exosomes to specifically target tumor cells or myocardial cells (Tian et al., 2014; Wang et al., 2018a). A study fused an MSC-binding peptide E7 with the lamp2b yields to prepare engineering exosomes, which had SMSC targeting capability. Specifically, plasmids encoding E7 were transferred into dendritic cells (DC) to produce DC-E7-exo through combining with lamp2b (Shao et al., 2012; Meng et al., 2015; Meng et al., 2017). Kartogenin (KGN) is a small molecule that has been discovered to induce differentiation of SMSC to chondrocytes both *in vitro* and *in vivo*. Therefore, KGN was loaded in DC-E7-exo by electroporation to prepare DC-E7-KGN-exo, which could efficiently enter SMSC and induce a higher degree of cartilage differentiation than KGN alone or KGN delivered by exosomes without E7, so it was a promising strategy to treat OA (Xu et al., 2021).

#### 3.2.3 Transcription factors

Nuclear factor I/C (NFIC) is a master transcription factor that’s critical for odontogenesis and osteogenesis, and deficiency of NFIC contributes to short root anomaly and dentin malformation. Based on previous findings that impaired odontoblastic differentiation was associated with a decline of NFIC in stem cells from apical papilla, researchers attempted to transfer and replenish NFIC by exploiting EVs, hopefully, providing a novel strategy for apical periodontitis therapy and hard tissue engineering. Human embryonic kidney cells 293 (HEK293FT cells) overexpressing NFIC protein were constructed by lentiviral transfection, and NFIC protein was loaded into vesicles by endogenous cell sorting mechanism to generate transgenic EVs overexpressing NFIC protein (HEK293FT-NFIC-EVs). HEK293FT-NFIC-EVs successfully upregulated NFIC levels in apical papilla, which promoted the proliferation, migration and odontogenic differentiation of apical papilla *in vitro* and the formation of dentin *in vivo* (Yang et al., 2022).

Scleraxis (Scx) is a helix-loop-helix transcription factor that can drive BMSC commitment to tenogenesis and has been reported that overexpression of Scx contributes to bone formation (Agarwal et al., 2017). Additionally, an increasing number of studies have found that Scx (+) and Sox9 (+) progenitors actively promoted tendon-bone healing, and deficiency of Scx expression impaired enthesis development (Blitz et al., 2013; Killian and Thomopoulos, 2016; Ideo et al., 2020). Exosomes from Scx-overexpressing BMSC (BMSC-Scx-exo) efficiently inhibited osteolysis and enhanced tendon-bone healing strength by preventing osteoclast formation. Exosomal RNA-seq revealed that the most highly expressed miRNA, miR-6924-5p, was a new miRNA in BMSC-Scx-exo. miR-6924-5p directly inhibited osteoclast formation by binding to the 3′-untranslated regions (3′UTRs) of OCSTAMP and CXCL12. Local injection of BMSC-Scx-exo or miR-6924-5p dramatically reduced osteoclast formation and improved tendon-bone healing strength (Feng et al., 2021).

#### 3.2.4 Other proteins

Glycoprotein non-melanoma clone B (GPNMB) is a multi-functional transmembrane glycoprotein expressed in numerous tissues, including bone (Rose et al., 2017). Growing evidence has shown that GPNMB is capable of regulating cell proliferation, adhesion, differentiation and extracellular matrix synthesis (Huang et al., 2016). In addition, GPNMB deficiency resulted in a reduction in the number of differentiated osteoblasts and the impairment of osteogenesis (Abdelmagid et al., 2014). Hence, GPNMB played a key role in osteoblast differentiation and bone homeostasis. EVs enriched with GPNMB (BMSC-GPNMB-EVs) were extracted from GPNMB-modified BMSC conditioned medium. BMSC-GPNMB-EVs significantly stimulated the proliferation and osteogenic differentiation of BMSC via the activation of Wnt/β-catenin signaling and attenuated the bone loss in the rat models of OP (Figure 2F) (Huang et al., 2021).

The current data indicate that osteogenic differentiation and impair ability of inflammation-compromised human periodontal ligament stem cells (PDLSC) could be reversed by P2X7 receptor (P2X7R) gene modification (Xu et al., 2019). PDLSC modified with P2X7R gene secreted transgenic exosomes expressing the P2X7R (PDLSC-P2X7R-exo). The microarray system showed that miR-3679-5p, miR-6515-5p and miR-6747-5p were highly expressed in PDLSC-P2X7R-exo, where miR-3679-5p and miR-6747-5p directly bound to Grem-1 protein, and miR-6515-5p indirectly bound to Grem-1 protein to rescue inflammatory-damaged PDLSC. In addition, PDLSC-P2X7R-exo also significantly promoted the osteogenesis of PDLSC through the PI3K-Akt-mTOR signaling pathway (Xu et al., 2020). It is evident that the proteins used to modify exosomes involve growth factors, targeting proteins, transcription factors, etc., which can achieve good bone regeneration and anti-inflammatory effects.

### 3.3 Genetically engineered exosomes modified with shRNA

Noggin is a natural BMP antagonist in response to BMP stimuli (Gazzerro et al., 1998). Studies have shown that the introduction of exogenous noggin impaired osteogenesis *in vitro* and bone formation *in vivo*, and inhibition of endogenous noggin augmented bone regeneration by activating endogenous BMP/Smad signaling (Fan et al., 2016). A convenient approach was created to collect and purify exosome mimetics (EMs) by extruding BMSC into polycarbonate membrane filters with progressively reduced pore size. Then, EMs were obtained from noggin-suppressed BMSC (BMSC-noggin-shRNA-EMs) and exhibited increased osteogenic properties. BMSC-noggin-shRNA-EMs encapsulated in an injectable chitosan hydrogel displayed substantial bone healing in rodent critical size calvarial defect models. Mechanically, elevated osteogenesis was mediated by increased noggin siRNA and decreased miRNA-29a (Fan et al., 2020).

The osteogenic induction of BMSC is mainly activated by the BMP/Smad signaling pathway (Salazar et al., 2016), however, SMURF1 may inhibit the BMP/Smad signaling pathway within the BMSC and further hinder their osteogenic differentiation (Popovic et al., 2014; Shimazu et al., 2016; Zheng and Shabek, 2017; Liang et al., 2018). Therefore, SMURF1-shRNA were transferred into the BMSC using viral vector to prepare engineering exosomes (BMSC-SMURF1-shRNA-exo), which were subsequently immobilized to the microarc oxide titanium implant surface with positively charged polyethyleneimine. The immobilized BMSC-SMURF1-shRNA-exo could be released slowly and consistently and subsequently phagocytosed by BMSC and macrophages, simultaneously activating the BMP/Smad signaling pathway in BMSC and promoting macrophage M2 polarization, both of which enhanced osseointegration. Thus, the microarc oxide titanium implants modified with BMSC-SMURF1-shRNA-exo provided a new method for promoting osteointegration between the prosthesis and host bone in revision total hip arthroplasty (X et al., 2022).

## 4 Engineering exosomes prepared by exogenous methods

### 4.1 Engineering exosomes modified with miRNA

It has been demonstrated the function of exosomal miRNA as intercellular signaling molecules. Among the thousands of miRNA genes, let-7 is one of the first to be discovered and its family members are highly conserved in sequence and function across various species. In animal models, let-7 acted as a key regulator of both normal development and cancer development (Su et al., 2012; Yang et al., 2013). Let-7 miRNA entered into exosomes from MC3T3-E1 cells by electroporation to obtain engineering exosomes (MC3T3-E1-let-7-exo), which restored the osteogenic capacity of MC3T3-E1 cells. At the same time, exosomes loaded with let-7 inhibitors by electroporation lost their osteogenic differentiation capacity. Therefore, MC3T3-E1-let-7-exo may play a crucial role in osteoblast differentiation and that exosomes whose let-7 miRNA were inactivated exhibited reduced differentiation activity (Choi et al., 2019). Milk-derived exosomes were utilized as a novel system for delivery of miR-31-5p and miR-31-5p mimics were successfully encapsulated into milk exosomes (milk-miR-31-5p-exo) through electroporation. Milk-miR-31-5p-exo had a higher rate of cell uptake and were able to resist degradation. Meanwhile, milk-miR-31-5p-exo significantly improved endothelial cell functions and enhanced the healing process of the diabetic wound by downregulating the expression of HIF1AN (Figures 4B–H) (Yan et al., 2022).

### 4.2 Engineering exosomes modified with proteins

BMP-2 were loaded directly into EVs derived from mouse J774A.1 mononuclear macrophages by electroporation or sonication to make engineering EVs (J774A.1-BMP2-EVs). The BMP-2 loading efficiency of sonication was 18% ± 1.9%, and the loading efficiency of electroporation was 5% ± 0.7%, which showed that sonication had a higher loading efficiency than electroporation. J774A.1-BMP2-EVs regulated the formation of osteoblasts and were free from protein degradation and noggin inhibition because they were located in the cavity. Moreover, the retention rate in the EVs is 100% for the first 24 h and 80% after 10 days. J774A.1-BMP2-EVs sent BMP-2 directly into the cytoplasm bypassing cell surface receptors, and then initiated BMP-2 signaling within the cell (Yerneni et al., 2021). The vascular endothelial growth factor (VEGF) is a crucial growth factor that has been shown to remodel the vasculature in many regeneration tissues (Martino et al., 2011; Choi et al., 2013). ATDC5 is a chondrogenic progenitor cell line that has been verified to exhibit significant osteogenic differentiation capacity (Yao and Wang, 2013). Hence, specifically engineering exosomes (ATDC5-VEGF-exo) have been constructed using ATDC5-derived exosomes to encapsulate plasmids carrying VEGF gene. ATDC5-VEGF-exo combined with 3D-printed porous bone scaffolds exhibited dual roles as an osteogenic matrix and a gene vector to potentially increase vascularized osteogenesis in segmental bone defects (Zha et al., 2021). In another research, ATDC5 were mechanically extruded to prepare EMs, and then loaded VEGF plasmids into EMs by electroporation to form engineering exosomes (ATDC5-VEGF-EMs). The coaxial electrospun nanofiber film of chitosan and poly lactic acid modified via Biotin-Avidin-System exhibited the high affinity to ATDC5-VEGF-EMs that eventually secreted VEGF to elevate vascularized osteogenesis *in situ* (Zha et al., 2020).

### 4.3 Engineering exosomes modified with siRNA

The bone-targeting peptides were anchored onto exosome membranes secreted by human induced pluripotent stem cells (iPS)-derived MSC, and then the siRNA of Shn3 were loaded in the exosomes by electroporation to form engineering exosomes (MSC-siShn3-exo). MSC-siShn3-exo have anti-osteoporotic function and the ability to specifically deliver siRNA to osteoblasts because bone-targeting peptides bound to periostin in a ligand-receptor specific manner for targeting osteoblasts. Silencing osteoblast Shn3 gene enhanced osteogenic differentiation, decreased autologous RANKL expression, prevented the development of osteoclasts as well as increasing the production of SLIT3, which promoted the formation of blood vessels, especially H-type blood vessels (Cui et al., 2022). Besides, FGF2, Wnt-11, S100A9, FSTL1, TNF-α, and Caspase3 siRNA were encapsulated into BMSC-exosomes by electroporation to produce BMSC-siRNA-exo. BMSC-siRNA-exo could effectively knock down these genes in vascular endothelial cells, significantly improving the proliferative ability of vascular endothelial cells and promoting ONFH repair through angiogenesis (Zhang et al., 2020a).

### 4.4 Engineering exosomes modified with PLGA

Recently, nanoparticle-based drug delivery systems have been developed for the targeting and treatment of rheumatoid arthritis (RA) with relatively reduced doses and side effects (Bosch, 2011; Hofkens et al., 2011). Cytochalasin B was applied to relax the interaction between the cytoskeleton and membrane of macrophages to secret MVs, which exhibited a similar bioactivity to that of RA-targeting macrophages. Poly (lactic-co-glycolic acid) (PLGA) nanoparticles were subsequently coated with MVs to prepare macrophage-derived MVs coated nanoparticles (MNP), and then, a model drug, tacrolimus, was encapsulated in MNP. The study demonstrated that the complicated MNP was an efficient biomimetic vehicle for RA targeting and significantly suppressed the progression of RA in mice (Figures 8A, B) (Li et al., 2019). Accumulating evidence has shown that a kind of 3D hydrogel composed of BMP-2 core sequence oligopeptides, phosphoserine, synthetic cell adhesion peptides and polyaspartic acid (PASP) has bone targeting properties and can synergistically promote bone regeneration by expediting the adhesion and proliferation of BMSC in rats (Quan et al., 2019). These studies provided the foundation for treating rotator cuff injury, combining the bone targeting properties of PASP with the carrier function of exosomes. BMP-2 was encapsulated to PASP-PLGA copolymer microcapsules firstly and then incubated with BMSC-exo to prepare engineering exosomes (BMSC-PASP-PLGA-BMP2-exo). In the end, these engineering exosomes released BMP-2 continuously showing good stability and targeting to BMSC and promoted tendon-bone healing after rotator cuff injury (Han et al., 2022).

## 5 Engineering exosomes prepared by chemical methods

### 5.1 Engineering exosomes modified with targeting peptide

**FIGURE 6:**
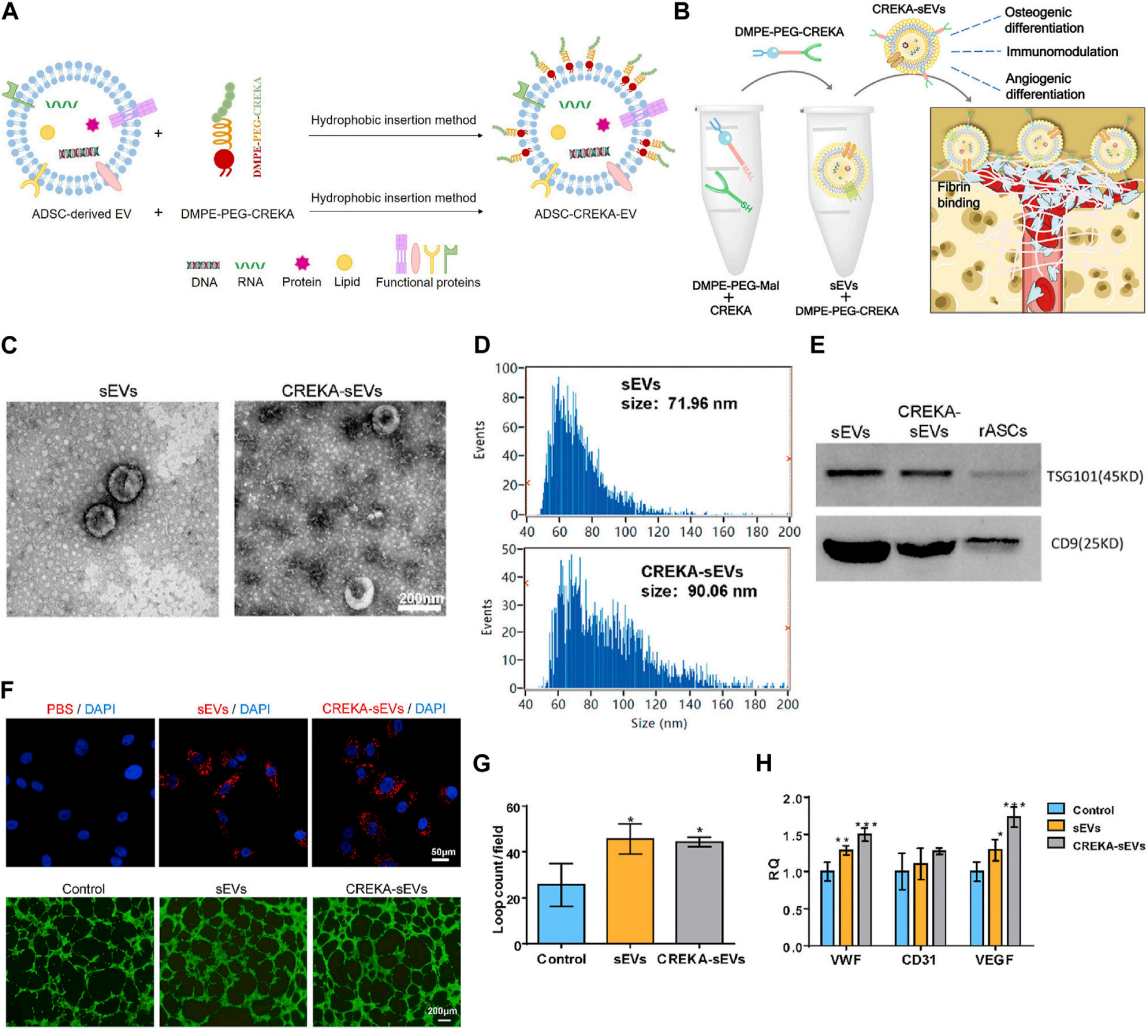
Hydrophobic insertion method to prepare CREKA functionalized EVs. **(A)** Process of hydrophobic insertion. **(B)** EVs modified with DMPE-PEG-CREKA can target fibrin, promote osteogenic differentiation of BMSC and angiogenic property of HUVEC, and modulate the polarization of macrophages (Wu et al., 2023). **(C)** TEM images of sEVs and CREKA-sEVs (Wu et al., 2023). **(D)** Particle size distribution of sEVs and CREKA-sEVs (Wu et al., 2023). **(E)** Western blotting analysis of TSG101 and CD9 (Wu et al., 2023). **(F)** Cellular internalization of CREKA-sEVs by HUVEC. Red: PKH26 labeled sEVs and CREKA-sEVs. Blue: Nuclei. *In vitro* tube formation of HUVEC (Wu et al., 2023). **(G)** Quantification of loop counts (Wu et al., 2023). **(H)** Key angiogenic gene expression of HUVEC (Wu et al., 2023).

Pentapeptide cysteine-arginine-glutamic acid-lysine-alanine (CREKA) exhibit a high affinity to fibrin–fibronectin complexes (Simberg et al., 2007), thereby is a promising bone defect targeting peptide. In a previous work, DMPE-PEG-CREKA were inserted into the membrane of EVs released from ADSC via the hydrophobic insertion method to obtain CREKA functionalized EVs (ADSC-CREKA-EVs), which could target fibrin and retain in bone defects (Figure 6A). The results also showed that ADSC-CREKA-EVs were able to promote the osteogenic differentiation of BMSC and the angiogenic property of HUVEC, and modulate the polarization of macrophages *in vitro*. Moreover, due to the improved fibrin-binding and retention capacity of ADSC-CREKA-EVs, they enhanced the bone repair substantially in the rat femoral defect models (Figures 6B–H) (Wu et al., 2023).

### 5.2 Engineering exosomes modified with aptamers

**FIGURE 7:**
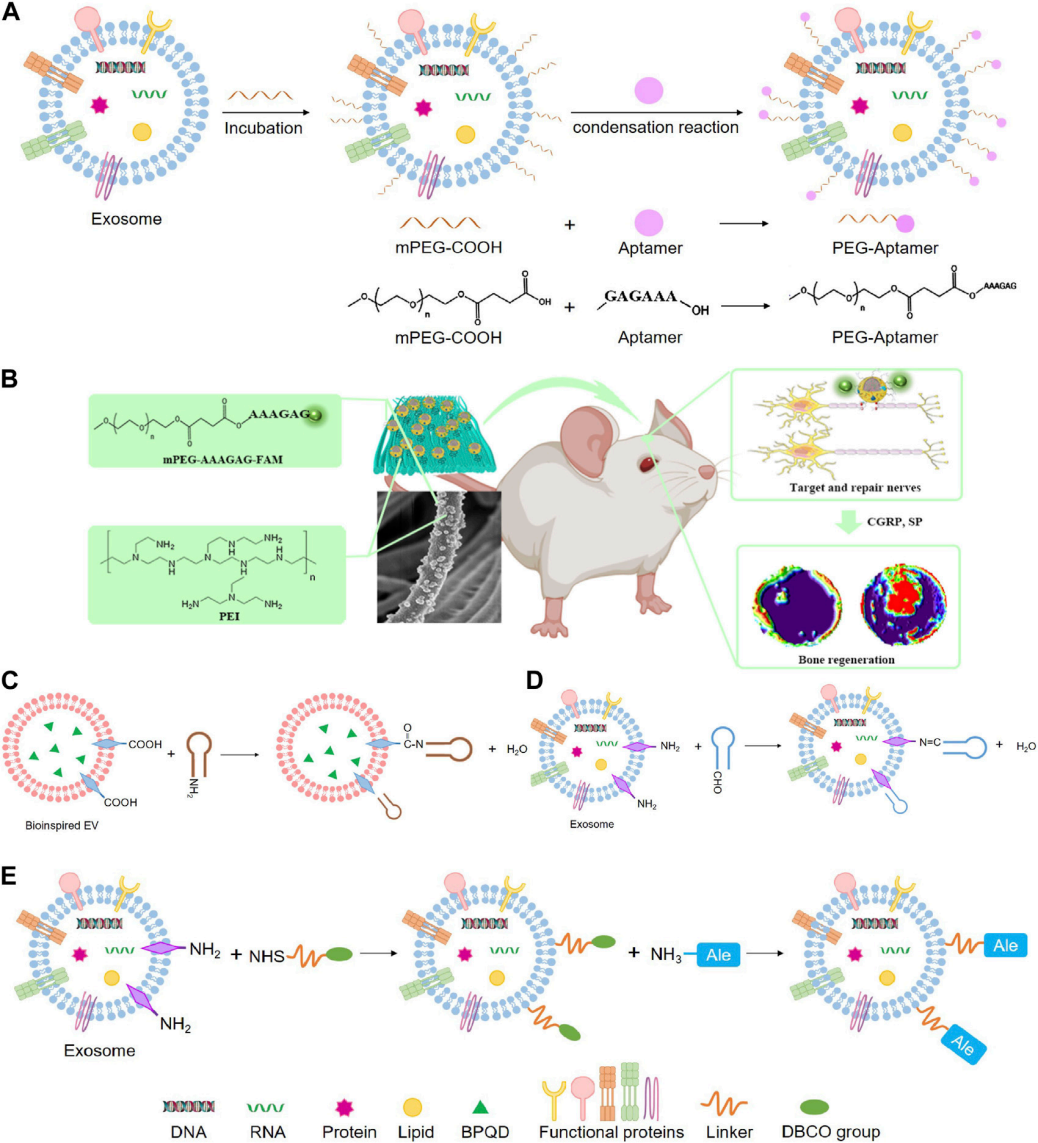
Chemical methods to prepare engineering exosomes. **(A)** Exosome modified with aptamers by condensation reaction. **(B)** The schematic diagram shows that PPEA electrospun biomimetic periosteum loaded with aptamers engineered exosomes can target injured axons and regenerate blood vessels and bone (Su et al., 2022). **(C)** Condensation reaction. **(D)** Schiff base reaction. **(E)** Click chemistry.

Recent studies have shown that phosphatidylserine is a lipid in cells exposed to the surface after axonal injury (Faris et al., 2021). Phosphatidylserine is essential in identifying and promoting axon fusion and regeneration after injury (Neumann et al., 2015). In an experiment, aptamers targeting phosphatidylserine were combined with exosomes from repair Schwann cells by self-assembly to construct engineering exosomes (Schwann-apt-exo) (Ashrafuzzaman et al., 2013). Then Schwann-apt-exo were successfully built on the surface of the electrospun fiber to prepare biomimetic periosteum, which could release exosomes in an acidic microenvironment and significantly induce nerve, blood vessel, and bone regeneration (Figures 7A, B) (Su et al., 2022). Black phosphorus quantum dots (BPQD) are ultrasmall nanosheets with sizes of several nanometers which have high photothermal conversion efficiency. Hyperthermia promotes biomineralization by stimulating the upregulated expression of proteins, including alkaline phosphatase and heat shock proteins (HSP) (Yanagi et al., 2015; Ota et al., 2017; Tong et al., 2019). Therefore, BPQD were encapsulated in PLGA nanoparticles to obtain the bioinspired EVs (Shao et al., 2016). Then, osteoblast-targeting aptamers were conjugated to bioinspired EVs (apt-EVs) for recognition of the osteoblasts in the bone region (Figure 7C). As a result, the bioinspired apt-EVs exhibited good selectivity and affinity to the rat osteoblasts and displayed outstanding bone regeneration performance (Wang et al., 2019). BMSC-derived exosomes are found to remarkably enhance osteoblastic differentiation of BMSC *in vitro*. However, intravenous injection of BMSC-exo is inefficient in ameliorating osteoporotic phenotypes in ovariectomy-induced postmenopausal OP mouse models, which may be exosomes are predominantly accumulated in the liver and lungs, but not in bone. Hereby, the BMSC-exo surface was conjugated with BMSC-specific aptamers (BMSC-apt-exo), which delivered exosomes into BMSC within bone marrow (Figure 7D). Intravenous injection of the BMSC-apt-exo complex enhanced bone mass in ovariectomy mice and accelerated bone healing in femur fracture mouse models. These results demonstrated the efficiency of BMSC-apt-exo in targeting bone to promote bone regeneration, providing a novel promising approach for the treatment of OP and fracture (Luo et al., 2019b).

### 5.3 Engineering exosomes modified with PLGA

**FIGURE 8:**
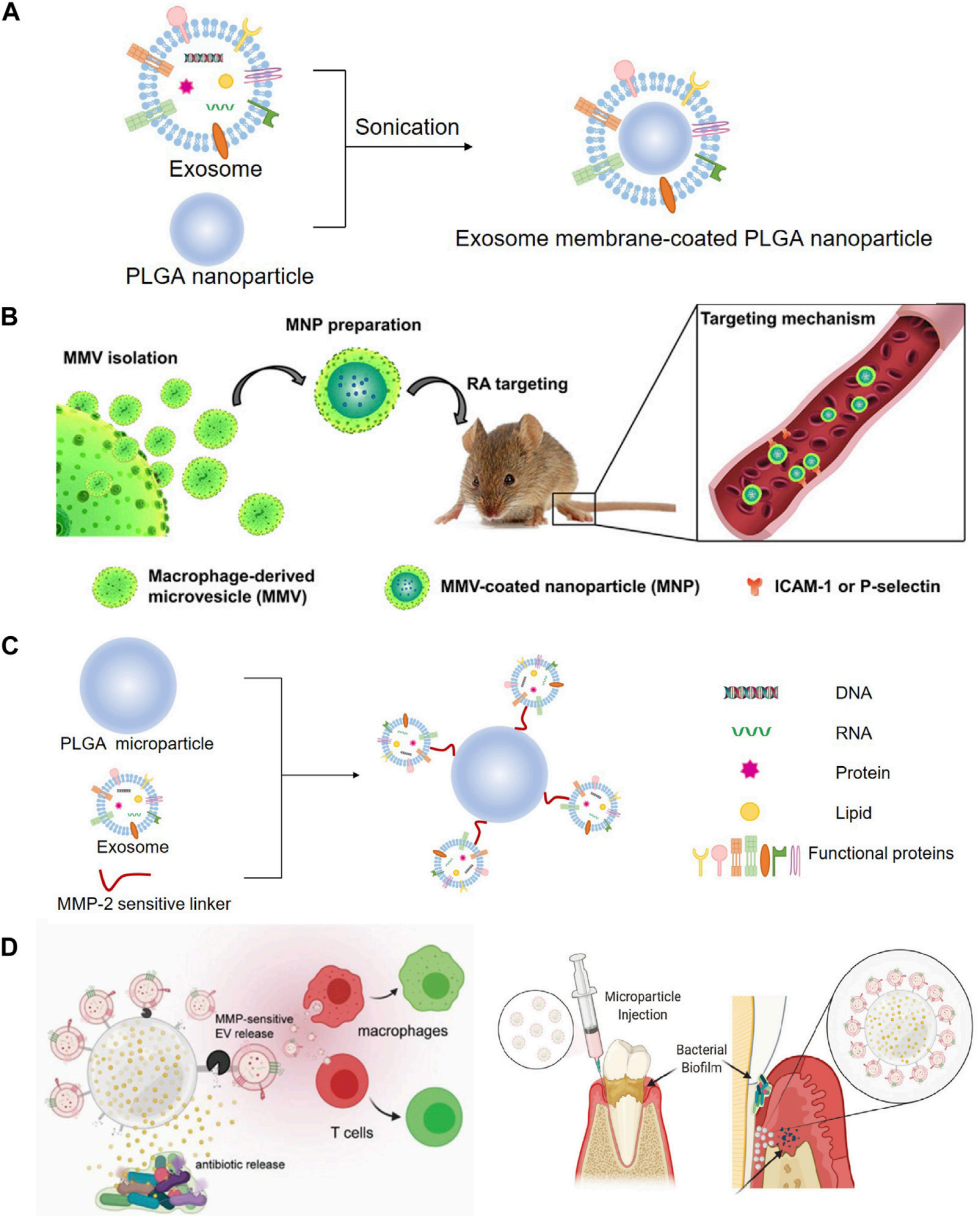
Modification with PLGA to prepare engineering exosomes. **(A)** Exosomes encapsulate PLGA nanoparticle by sonication. **(B)** Schematic illustration of MMV-coated nanoparticle (MNP) targeting sites of RA. MNP could target sites of RA through ICAM-1 or P-selectin adhesion (Li R. et al., 2019). **(C)** EVs derived from GMSC were immobilized on the surface of the PLGA via a MMPs-sensitive linker, **(D)** enabling the targeted release of the EVs by MMPs at the site of inflammation for localized immunomodulation (Zarubova J. et al., 2022).

A study developed a dual delivery platform based on PLGA releasing antibiotics to suppress bacterial biofilm growth. Meanwhile, EVs derived from gingival mesenchymal stem cells (GMSC) were immobilized on the surface of the microparticles via a matrix metalloproteinases (MMPs)-sensitive linker, enabling the targeted release of the EVs by MMPs at the site of inflammation for localized immunomodulation. GMSC-EVs were able to decrease the secretion of pro-inflammatory cytokines by monocytes/macrophages and T cells, suppress T cells activation, induce the formation of regulatory cells *in vitro* and significantly improve regeneration of the damaged periodontal tissue in rat models of periodontal disease (Figures 8C, D) (Zarubova et al., 2022).

### 5.4 Engineering exosomes modified with other substances

Dextran sulfate (DS) consists of linear 1,6-linked glucose pyran with sulfate groups, which is able to target the abundant macrophage scavenger receptor A (SR-A) at the inflammatory joints of RA (Heo et al., 2017), thus constructing engineered ADSC-derived exosomes (ADSC-DS-exo) by metabolic glycoengineering-mediated click chemistry. ADSC-DS-exo targeted and activated macrophages accumulating in inflamed joints, and efficiently induced a cascading of anti-inflammatory responses by modulating macrophage phenotypes (You et al., 2021). Alendronate (Ale) specifically targets bone tissue via hydroxyapatite. Therefore, mouse MSC-derived EVs were combined with Ale to generate MSC-Ale-EVs by click chemistry to facilitate EVs targeting bone via Ale/hydroxyapatite binding. The MSC-Ale- EVs had a high affinity with bone and had great potential for clinical applications in OP therapy with low systemic toxicity (Figure 7E) (Wang et al., 2020).

## 6 Discussion

Some findings have suggested that the transplantation of gene-modified stem cells lead to an improved therapeutic outcome. However, the large-scale use of gene-transferred cells in the clinic is associated with additional safety concerns. In fact, the direct introduction of exogenous stem cells has several deficiencies, such as reduced bioactivity, reject reaction, and regional tumorigenesis, pathological changes and chromosomal variation (Herberts et al., 2011; Brennan et al., 2020). Besides, the local inflammatory environment not only impairs the therapeutic efficacy of transplanted cells but also negatively affects the regenerative potential of resident stem cells. On the other hand, compared with the direct administration of medicines, nanocarrier-based therapy has many superiorities (Li et al., 2022), such as encapsulation of hydrophobic drugs, biomacromolecule immobilization, enzymes protection, immune system escape and cellular uptake of through endocytosis (Tesarova et al., 2020). Therefore, it is important to develop a superior strategy which can fully utilize the advantages of nanocarriers and prevent the potential risks of stem cell transplantation.

In recent decades, synthetic nanoparticles including liposomes, micelles, dendrimers, nanocapsules, nanodiamonds, nanosponges, nanoemulsions, and self-assembled peptides have been extensively studied for nanomedicine, particularly for targeted therapy and drug delivery (Li et al., 2021). However, exogenous nanomaterials for delivering drugs to the target site face many hurdles, such as immunogenicity, rapid clearance and various biological barriers (Blanco et al., 2015). One approach to overcome the limitations of synthetic nanomaterials is to develop natural carriers.

The cell-free tissue engineering has been recognized as a safe and effective strategy in the field of regenerative medicine. Accumulating evidence has indicated that the therapeutic effect of stem cell transplantation is mediated through exosome secretion (Syn et al., 2017). Exosomes transmit bioactive proteins, lipids, DNA and RNA to target cells to protect cargos inside from RNases and phagocytosis, and they also have intrinsic homing effect, high stability in circulation, efficient intercellular communication, low tumorigenicity and immunogenicity and excellent immune stealth capacity (Kourembanas, 2015). Some MSC-EV human clinical trials have been conducted, it’s reported that over 80 studies have been registered at the www.ClinicalTrials.gov database to assess the therapeutic effects of EVs in several therapeutic areas (Johnson et al., 2021). For example, a patient with severe therapy-refractory acute graft-versus-host disease has considerable improvement after repeated injections of MSC-EVs, and the patient’s pro-inflammatory cytokine levels and illness symptoms reduced significantly (Kordelas et al., 2014). In another Phase II/III placebo-controlled clinical trial, MSC-EVs derived from umbilical cord blood modulated inflammation and improved kidney function in patients with chronic kidney disease (Nassar et al., 2016). In addition to these early-phase clinical trials, several trials of therapeutic EV-products are ongoing by some biopharmaceutical companies, reflecting the increasing maturity of the therapeutic EV field, such as platelet-derived EVs tested by Exopharm in two Phase I clinical trials for wound healing (Johnson et al., 2021).

Despite exosomes have great potential in therapeutic delivery, exosomes still have shown limited application in clinical studies, such as low targeting, low yield and low therapeutic effect (Sun et al., 2016; Colao et al., 2018). Besides, use of exosomes alone was not adequate for complete tissue regeneration, especially in challenging environments (e.g., large skeletal defects) due to their limited inductive capacity (Pegtel et al., 2010; Alvarez-Erviti et al., 2011; Sterzenbach et al., 2017). In order to overcome the shortcomings, increasing studies have taken measures to improve the properties by modifying the exosomes. On one hand, exosomes derived from genetically modified cells exhibit enhanced bone regeneration potential and have the potential to be used as future therapeutic strategies. It is relatively simple to regulate exosome content by manipulating donor cells, and the structural integrity of exosomes is not adversely affected, which is conducive to maintaining their function. Enhanced expression of specific genes in MSC can upregulate the corresponding exosome cargos, thereby inducing prolonged therapeutic effect. But there’s a problem that viral transduction of host cells to overexpress miRNA or proteins has a possibility of potential safety risks. Moreover, preparing genetically engineered exosomes using endogenous methods is more difficult and takes longer than using exogenous methods. On the other hand, extra inductive or therapeutic factors like small molecule drugs and siRNA have been loaded in exosomes by exogenous methods to enhance exosome-based tissue regeneration and skeletal disease treatment. Meanwhile, engineering exosomes produced by exogenous methods have higher stability between batches and are more suitable for large-scale production. But the less satisfying part is that the exogenous transport of these cargos raises concerns about loading rate and potential adverse effects. The packaging efficiency of different loading methods differs significantly, so it’s necessary to explore how to get maximum load efficiency later. Additionally, exosome membranes may be damaged during the loading process, and the consequences on the human body are unknown.

Many researchers use chemical techniques to modify exosomes to enhance biological function and improve delivery efficiency. It is undoubtedly very important to solve the problem of low targeting of exosomes for clinical application. A lot of different strategies have been employed for construction of targeting delivery systems. For example, several researchers modified exosomes with multiple functional components, such as targeted peptides or aptamers (Nakagawa et al., 2010; Ara et al., 2014; Sun et al., 2016). However, biochemical conjugation might alter the natural characteristics and functions of the engineering exosomes and complex conditions *in vivo* might weaken their targeting properties so that risks of potential off-target effects still exist.

Frankly speaking, these engineering exosomes seem inadequate for large-scale clinical-grade manufacturing. Therefore, some researchers have started to create new methods to prepare exosome mimics, such as producing exosome mimics by extruding cells or microfluidic devices (Jang et al., 2013; Jo et al., 2014; Yoon et al., 2015; Fan et al., 2020), and then modify these exosome mimics to prepare engineering exosomes. The emergence of exosome-mimetics greatly promotes the development of conventional exosome-based gene therapy as it successfully addresses the major concern of low yield. But the difference between these mimics and natural exosomes is still unknown and their long-term stability and safety require further improvement.

We have to admit that quality control and storage of engineering exosomes are significant challenges. The quality control during the production process must be carefully monitored to ensure the stability between batches and prevent bacterial contamination. The biological structure of exosomes themselves is as important as the cargo they carry (Jiang et al., 2022). Therefore, in addition to protecting the structure of exosomes, it is also necessary to prevent leakage or destruction of therapeutic cargos. At present, the most commonly used preservation techniques are freezing, freeze-drying and spray-drying (Zhang et al., 2020). Cryopreservation is typically used at temperatures of 4°C, 80°C, and 196°C, but this storage method is prone to “frostbite”. In order to overcome this shortcoming, different kinds of antifreezes need to be added to extend the shelf life, such as dimethyl sulfoxide, ethylene glycol, trehalose, sucrose and so on (Jeyaram and Jay, 2017; Bahr et al., 2020). The exosome can retain its original activity during freeze-drying because the dehydration and drying of the product occurs at low temperature and under vacuum condition, however, the molecular structure of the biomolecule may be destroyed due to the freezing and dehydration pressures generated. Spray-drying means that the EVs solution is atomized in a drying chamber and the moisture is quickly evaporated in contact with hot air to produce dry powders. The stability of exosomes during the process is influenced by the outlet temperature and atomization pressure (Kusuma et al., 2018). Unfortunately, no matter which storage technique is more or less problematic, especially when attempting to preserve exosomes for a long time, so it’s essential to find more suitable ways to store exosomes and study the activity and function of exosomes after preservation.

## 7 Conclusion

Genetically or chemically engineered exosomes provide a promising strategy for treating bone or cartilage injury and inflammation-related diseases. These engineering exosomes can be produced by various methods, such as endogenous, exogenous and chemical methods. However, the translation of engineering exosomes for clinical applications requires further research about safety, feasibility and mass productivity. In the future, researchers need to improve packaging efficiency, explore new substances that can modify exosomes to serve tissue engineering better as well as ensure mass production of engineering exosomes scalable, stable and efficient.

## Figures and Tables

**Table:** 

**RA**	rheumatoid arthritis
**OP**	osteoporosis
**OA**	osteoarthritis
**ONFH**	osteonecrosis of the femoral head
**EVs**	extracellular vesicles
**Exo**	exosomes
**MVs**	microvesicles
**TEM**	transmission electron microscope
**MSC**	mesenchymal stem cells
**Cur**	curcumin
**miRNA**	MicroRNA
**BMSC**	bone marrow mesenchymal stem cells
**HUVEC**	human umbilical vein endothelial cells
**MMP14**	matrix metalloproteinase 14
**SMSC**	synovial mesenchymal stem cells
**ADSC**	adipose-derived stem cells
**BMP**	bone morphogenetic protein
**NELL1**	Neural epidermal growth factor-like 1
**3D**	three-dimensional
**CXCL12**	Chemokine C-X-C motif ligand 12
**CXCR4**	chemokine C-X-C motif receptor 4
**GLG1**	Golgi glycoprotein 1
**TNFR1**	tumor necrosis factor receptor 1
**IL-6ST**	interleukin-6 signal transducer
**TNF-α**	tumor necrosis factor alpha
**Lamp2b**	lysosome-associated membrane glycoprotein 2b
**DC**	dendritic cells
**KGN**	Kartogenin
**NFIC**	Nuclear factor I/C
**Scx**	Scleraxis
**GPNMB**	glycoprotein non-melanoma clone B
**PDLSC**	periodontal ligament stem cells
**P2X7R**	P2X7 receptor
**EMs**	exosome mimetics
**VEGF**	vascular endothelial growth factor
**iPS**	induced pluripotent stem cells
**CREKA**	cysteine-arginine-glutamic acid-lysine-alanine
**BPQD**	black phosphorus quantum dots
**HSP**	heat shock proteins
**PLGA**	poly(lactic-co-glycolic acid)
**ZF**	zinc finger
**MNP**	MVs coated nanoparticles
**PASP**	polyaspartic acid
**GMSC**	gingival mesenchymal stem cells
**MMPs**	matrix metalloproteinases
**DS**	dextran sulfate
**SR-A**	scavenger receptor A
**Ale**	Alendronate
